# Comparative effectiveness of culturally adapted oral health education in rural Quechua-speaking schoolchildren: a cluster quasi-experimental study

**DOI:** 10.3389/froh.2026.1903080

**Published:** 2026-07-31

**Authors:** Vilma Mamani-Cori, Franz A. Jamachi-Ccama, Evelyn L. Tintaya-Quispe, José M. Aguilar-Huanca, Marco A. Quispe-Apaza, Betsy Quispe-Quispe, Tania C. Padilla-Caceres, Yessica Quilca-Soto, Naysha S. Villanueva-Alvaro, Claudia J. Lauracio-Lope

**Affiliations:** 1 Oral Health Researchers Team (OHRT), School of Dentistry, Faculty of Health Sciences, Universidad Nacional del Altiplano, Puno, Peru; 2 School of Dentistry, Faculty of Health Sciences, Universidad Nacional del Altiplano, Puno, Peru

**Keywords:** cultural adaptation, health literacy, indigenous peoples, oral health education, Quechua people, rural schoolchildren

## Abstract

**Background:**

Oral health disparities remain prevalent among Indigenous and rural populations, where linguistic barriers and limited access to culturally appropriate health education may reduce the effectiveness of conventional oral health promotion strategies. Although school-based oral health education programs have demonstrated beneficial effects, evidence on culturally adapted educational modalities for Indigenous-language-speaking communities remains limited.

**Objective:**

To evaluate the effectiveness of culturally adapted oral health educational interventions delivered in Quechua and Spanish through audio and video modalities on oral health knowledge, attitudes, and behaviors among rural schoolchildren from Quechua-speaking communities in the Peruvian Andes.

**Materials and methods:**

A cluster quasi-experimental study with four parallel intervention groups and repeated measures was conducted among 227 schoolchildren aged 8–12 years from two rural public schools in Puno, Peru. Classroom sections were allocated to Quechua- or Spanish-language instruction, and students within each classroom were randomly assigned to receive audio- or video-based educational materials, resulting in four intervention groups: Spanish-Audio (*n* = 55), Quechua-Audio (*n* = 58), Spanish-Video (*n* = 60), and Quechua-Video (*n* = 54). Knowledge, attitudes, and behaviors were assessed at baseline, immediately after the intervention, and at 24-hour and 7-day follow-ups using the Measurement of Knowledge, Attitudes, and Behaviors Regarding Oral Health in Schoolchildren Scale (MKAB-OH-S). Data were analyzed using Chi-square, Friedman, Wilcoxon signed-rank, Kruskal–Wallis, and Mann–Whitney *U*-tests.

**Results:**

All intervention modalities significantly improved oral health knowledge, attitudes, and behaviors over time (all *p* < 0.001). Between-group differences were observed only for knowledge immediately after the intervention (*p* < 0.001) and at the 24-hour follow-up (*p* = 0.032). The Spanish-Audio group showed lower knowledge scores than the other intervention groups during the early post-intervention assessments. The largest longitudinal effect sizes were observed for knowledge, particularly in the Quechua-language intervention groups.

**Conclusions:**

Culturally and linguistically adapted oral health education supported by audiovisual resources improved oral health knowledge, attitudes, and behaviors among rural Quechua-speaking schoolchildren. These findings support the incorporation of culturally appropriate educational strategies into school-based oral health promotion programs to strengthen prevention and help reduce oral health inequalities in underserved Indigenous communities.

## 1 Introduction

Oral diseases continue to be a frequent public health problem in different regions of the world. Among them, untreated dental caries stands out due to its high prevalence and because it affects people of all ages. According to the World Health Organization, this disease affects billions of people and remains among the most common chronic conditions during childhood (1). Its consequences are not limited to the oral cavity, as they can impact children's nutrition, school performance, quality of life, and psychosocial well-being (2–5). This situation is often more pronounced in socially vulnerable groups, especially in rural and indigenous communities where access to preventive services and adequate oral health information remains limited (6, 7).

Given this scenario, schools have become important spaces for developing oral health promotion activities. From an early age, educational interventions aim to strengthen knowledge and foster habits that contribute to the prevention of oral diseases (8). Several systematic reviews have shown favorable results in terms of knowledge, attitudes, and preventive behaviors among schoolchildren participating in these types of programs (9–11). However, the observed effects are not the same in all contexts. Differences related to language, local culture, the way the content is presented, and the level of community participation could explain some of this variability.

Among the factors that can influence the success of educational interventions, language plays a significant role. Effective communication is a fundamental component of any health promotion strategy, and difficulties can increase when information is conveyed in a language other than the one usually used by the participants. Previous studies have indicated that language barriers can hinder the understanding of health messages, decrease participation in educational activities, and contribute to the perpetuation of inequalities in indigenous populations (12–16). Conversely, information presented in a culturally familiar language can be more understandable and generate greater trust. Research conducted in indigenous contexts has also shown that the preservation of native languages and the use of culturally appropriate communication strategies are associated with greater community participation and more favorable health outcomes (17).

These considerations are especially important in the Peruvian context, characterized by its broad linguistic and cultural diversity. Quechua is the most widely spoken indigenous language in the country and remains the primary means of communication in numerous rural Andean communities. Although policies promoting interculturality in health have been implemented in recent decades, a large proportion of educational and preventative materials continue to be developed primarily in Spanish. As a result, many Quechua-speaking children receive health information in a language different from that used in their family and community (18). Several studies have highlighted the need to incorporate the Quechua language and local cultural perspectives into oral health care and education activities in order to reduce communication barriers and bring services closer to indigenous communities (12, 18). However, empirical evidence directly comparing educational interventions developed in Quechua and Spanish remains limited, particularly among schoolchildren in rural areas.

In addition to language, the way educational content is presented can also influence learning. In recent years, audiovisual resources have been increasingly incorporated into health education activities due to their ability to combine visual and auditory stimuli. Educational videos, animated characters, and narrative-based materials have shown favorable results in various health education contexts aimed at children and adolescents (19, 20). These types of resources can facilitate understanding of messages, capture attention for longer periods, and promote the retention of information relevant to health care.

Among these strategies, storytelling or story-based learning has sparked increasing interest. The use of narratives allows preventive messages to be presented in a way that is more relatable and understandable for children, integrating information within situations they can easily recognize or imagine. In addition to increasing interest in the content, narratives can help information be remembered more easily. In rural and culturally diverse contexts, stories that incorporate elements close to the reality of schoolchildren could be a useful tool for strengthening learning processes and making educational messages more meaningful (21).

Despite growing interest in culturally adapted health education and audiovisual learning strategies, evidence remains limited in several respects. Most oral health education studies have been conducted in urban populations, have focused on dominant-language groups, or have evaluated a single educational modality. As a result, little is known about the comparative effectiveness of Indigenous-language interventions among rural schoolchildren or about the extent to which language and delivery format may influence oral health learning outcomes. This evidence gap is particularly evident in Quechua-speaking populations, despite the large number of children living in bilingual and intercultural environments throughout the Peruvian Andes.

Generating evidence in these contexts is essential for the development of equitable and culturally responsive oral health promotion programs. Therefore, this study aimed to evaluate the effectiveness of culturally adapted oral health educational interventions delivered in Quechua and Spanish through audio and video modalities on knowledge, attitudes, and behaviors among rural schoolchildren from Quechua-speaking communities in the Andean region of Peru.

## 2 Materials and methods

### 2.1 Study design and setting

A cluster quasi-experimental study with four parallel educational intervention groups and repeated measures was conducted between April and June 2025 in two rural public primary schools located in Capachica, Puno, Peru. The study evaluated the comparative effectiveness of culturally adapted audiovisual oral health education modalities delivered in Quechua and Spanish among schoolchildren from Quechua-speaking communities.

The intervention groups were organized according to the educational language and delivery modality as follows: Spanish-audio, Quechua-audio, Spanish-video, and Quechua-video. Outcome assessments were performed at baseline, immediately after the intervention, at the 24-hour follow-up, and at the 7-day follow-up.

### 2.2 Participants and sampling

The study population consisted of 278 schoolchildren aged 6–13 years enrolled in the participating schools. Children aged 8–12 years who met the eligibility criteria were considered for inclusion. Thirty-eight schoolchildren were excluded prior to allocation because they did not meet the eligibility criteria, including age outside the predefined range (*n* = 34) or temporary residence outside the local rural communities (*n* = 4).

The inclusion criteria were: (1) regular attendance at the participating primary schools; (2) signed informed consent from parents or legal guardians; (3) child assent to participate; (4) ability to speak and understand Quechua; and (5) absence of severe systemic disease that could prevent participation during the intervention period.

Participants who were absent during one or more follow-up assessments were not included in the final longitudinal analysis.

Sample size estimation was based on detecting moderate effect sizes in repeated-measures comparisons among four intervention groups, considering an effect size of f = 0.25, a significance level of *α* = 0.05, and a statistical power of 80%. Considering potential participant attrition during follow-up assessments, an initial target of 60 participants per group was established.

Classroom sections served as the clusters for group allocation. In each school, one section (A or B) received the educational materials in Quechua, whereas the other received the Spanish version. Within each language group, children were subsequently assigned to either the audio or video modality through a simple lottery procedure. At the start of the study, 60 participants were included in each intervention arm, resulting in a total sample of 240 eligible schoolchildren. Some participants missed one or more follow-up assessments and were therefore excluded from the longitudinal analyses. Consequently, the final analytical sample comprised 227 children distributed across the four intervention groups as follows: Spanish-audio (*n* = 55), Quechua-audio (*n* = 58), Spanish-video (*n* = 60), and Quechua-video (*n* = 54).

Although classroom sections served as the clustering unit for language allocation, the educational modality (audio or video) was assigned individually within each classroom through simple random allocation. Consequently, the study combined cluster-level allocation for language with individual-level allocation for educational modality, rather than assigning all four intervention groups by cluster.

### 2.3 Educational intervention

The intervention was based on culturally adapted audio and video materials covering four oral health topics: dental caries, oral bacteria, healthy eating habits, and toothbrushing practices. To make the content more engaging for children, the materials incorporated storytelling elements and animated characters adapted to the target age group.

The same educational script was used for both formats, with the only difference being the mode of delivery. According to group allocation, participants received the material in either Spanish or Quechua. The Quechua version was adapted by bilingual collaborators familiar with the local language variant spoken in the study area.

The duration of the materials was approximately 4.5 min in Spanish and 5 min in Quechua. Educational sessions were conducted collectively in classrooms or school auditoriums using audio equipment and multimedia projectors. Each group received the assigned material once a day for seven consecutive school days between 9:00 and 11:00 a.m.

Because the study instrument included items related to dental attendance, children were also provided with brief information about dental visits. In all intervention groups, participants were informed that attending a dental check-up at least once a year was recommended. This information was communicated in the language corresponding to each intervention group.

Before implementation, the educational materials were reviewed by specialists in pediatric dentistry and by a Quechua-speaking educator. Their evaluation focused on content relevance, clarity of the messages, cultural appropriateness, and suitability for the intended audience.

### 2.4 Instrument

Information was gathered using the Measurement of Knowledge, Attitudes, and Behaviors Regarding Oral Health in Schoolchildren Scale (MKAB-OH-S), a validated instrument developed for use with rural schoolchildren (22). The questionnaire assesses three areas related to oral health: knowledge, attitudes, and behaviors.

The knowledge section includes five multiple-choice questions. Each correct answer receives one point and incorrect answers receive zero points, producing a total score between 0 and 5.

Attitudes and behaviors are assessed through two separate sets of five items. Responses are recorded on a five-point Likert-type scale represented by facial expressions that facilitate understanding among children. For both domains, possible scores range from 5 to 25, with higher values reflecting more favorable attitudes and healthier oral health behaviors.

The MKAB-OH-S had previously undergone psychometric evaluation in a comparable population of rural Andean schoolchildren. The instrument demonstrated acceptable construct validity, substantial inter-rater reliability (Cohen's *κ* = 0.83), and satisfactory internal consistency, with Cronbach's alpha coefficients of 0.65 for knowledge, 0.91 for attitudes, and 0.73 for behaviors. Therefore, no additional pilot reliability assessment was conducted as part of the present intervention study.

### 2.5 Data collection procedure

Baseline assessments were conducted before the first educational intervention session. Immediately after the first intervention, participants completed the post-test assessment. Follow-up evaluations were subsequently conducted at the 24-hour follow-up and the 7-day follow-up after the initial intervention. Follow-up assessments were performed before the educational session scheduled for the corresponding evaluation day.

The questionnaire was self-administered under researcher supervision. Instructions and guidance were provided in the same language assigned to the intervention group, and participant questions were answered in the corresponding language. The average completion time for the questionnaire was approximately 10 min.

Data collection and intervention procedures were performed by senior dental students previously trained and standardized for educational delivery and questionnaire administration. Prior to data collection, investigators participated in training sessions to standardize participant instructions, intervention delivery, the brief complementary recommendation regarding annual dental visits, and questionnaire administration procedures. Investigators assigned to the Quechua-language groups were fluent in both Quechua and Spanish.

### 2.6 Ethical considerations

The study was approved by the Ethics Committee of the Universidad Nacional del Altiplano (N° 080/CIE UNA-Puno). Authorization to conduct the study was obtained from the administrative authorities of the participating schools.

Written informed consent was obtained from parents or legal guardians, and written assent was obtained from all participating children prior to data collection. The study followed the ethical principles established in the Declaration of Helsinki for research involving human participants.

The study protocol was reviewed and approved by the Institutional Research Ethics Committee before participant recruitment. As this was a school-based quasi-experimental educational intervention rather than a clinical trial, the study was not prospectively registered in a public trial registry.

### 2.7 Statistical analysis

Statistical analyses were performed using IBM SPSS Statistics version 27.0 (IBM Corp., Armonk, NY, USA).

Participants who did not complete one or more follow-up assessments were excluded from the corresponding longitudinal analyses (complete-case analysis). Because the proportion of missing follow-up data was low and losses occurred after baseline assessment, no data imputation, sensitivity analyses, or intention-to-treat analyses were performed.

Language allocation was performed at the classroom level, whereas the educational modality was randomly assigned to individual students within each classroom. Consequently, the study followed a partially clustered design rather than a conventional cluster trial in which each intervention arm corresponds to an independent cluster. Only four classroom clusters (two schools with two classroom sections each) were available. Given this very small number of clusters, multilevel models or generalized estimating equations were not applied because reliable estimation of between-cluster variance and cluster-level standard errors generally requires a substantially larger number of clusters (23, 24). Accordingly, analyses were conducted at the individual level using nonparametric methods.

Categorical variables were summarized as frequencies and percentages, whereas continuous and ordinal variables were described using medians and interquartile ranges (IQRs), as data did not meet normality assumptions.

Baseline sociodemographic characteristics were compared across intervention groups using the chi-square test for categorical variables and the Kruskal–Wallis test for continuous or ordinal variables.

Longitudinal changes in knowledge, attitudes, and behaviors within each intervention group were evaluated using the Friedman test. When significant overall differences were detected, pairwise *post hoc* comparisons were performed using the Wilcoxon signed-rank test with Bonferroni correction.

Between-group comparisons at each assessment time point were conducted using the Kruskal–Wallis test. When statistically significant differences were identified, pairwise comparisons were performed using the Mann–Whitney *U*-test with Bonferroni adjustment.

Effect sizes for longitudinal changes were estimated using Kendall's coefficient of concordance (Kendall's W) derived from the Friedman test. Values of approximately 0.10, 0.30, and 0.50 were interpreted as small, moderate, and large effects, respectively. For pairwise between-group comparisons, effect sizes were calculated as r = |Z|/√N and interpreted according to conventional thresholds (0.10 = small, 0.30 = moderate, and 0.50 = large).

A two-sided *p*-value < 0.05 was considered statistically significant unless otherwise specified by Bonferroni-adjusted comparisons.

## 3 Results

### 3.1 Participant flow

A total of 240 schoolchildren met the eligibility criteria, provided consent/assent, and were allocated to four intervention groups (*n* = 60 per group): Spanish-Audio, Quechua-Audio, Spanish-Video, and Quechua-Video. Outcome assessments were conducted at baseline, immediately after the intervention, at the 24-hour follow-up, and at the 7-day follow-up.

**Figure 1:**
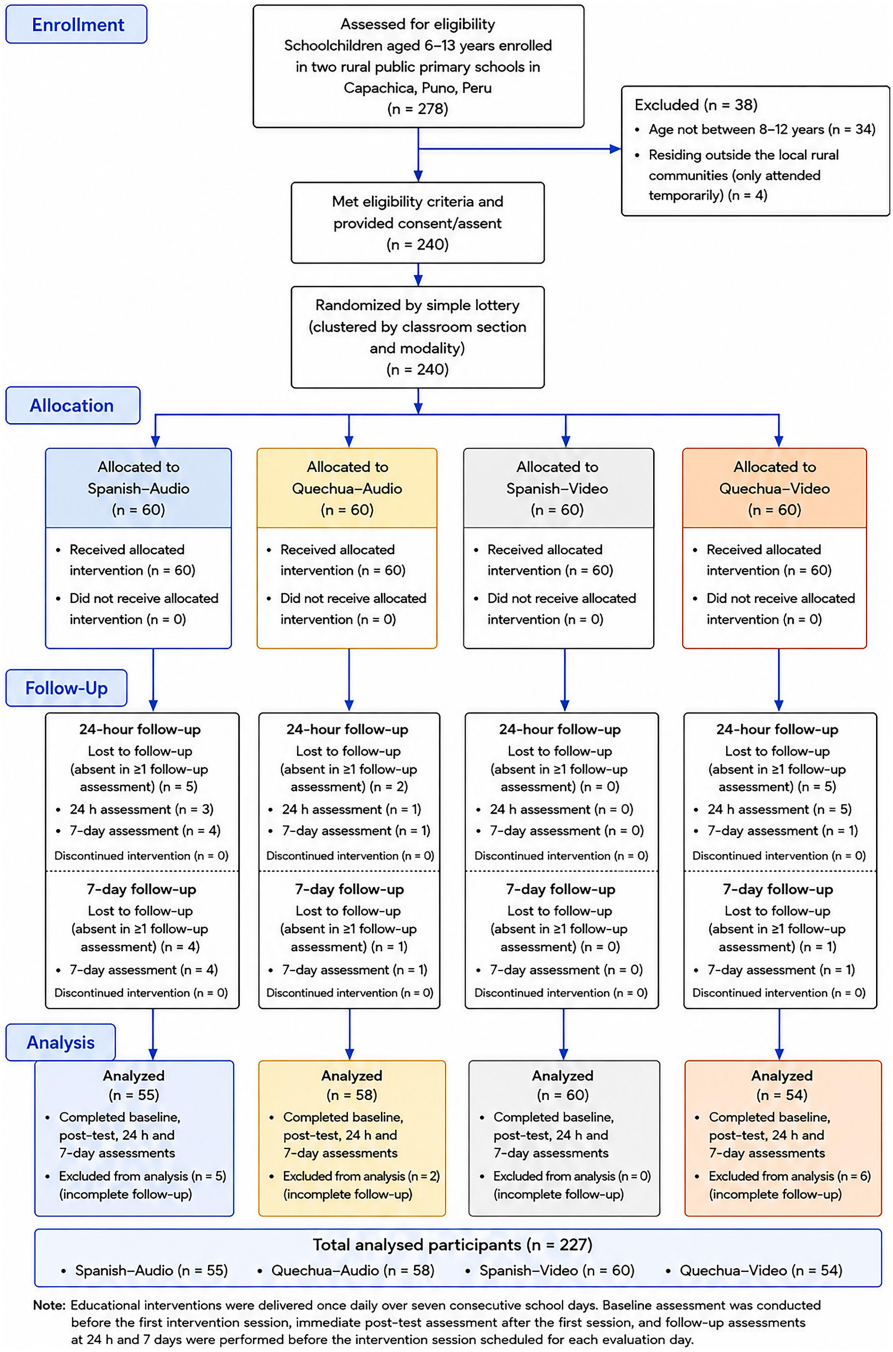
CONSORT flow diagram.

During follow-up, some participants were absent from one or more scheduled assessments and were therefore excluded from the final longitudinal analysis. The final analytical sample comprised 227 participants: Spanish-Audio (*n* = 55), Quechua-Audio (*n* = 58), Spanish-Video (*n* = 60), and Quechua-Video (*n* = 54). The flow of participants through the study is presented in Figure 1.

### 3.2 Baseline characteristics

The baseline sociodemographic characteristics and outcome measures of the participants are presented in Table 1. No significant differences were observed among intervention groups regarding age, sex distribution, maternal education level, or number of siblings (all *p* > 0.05). Baseline knowledge, attitude, and behavior scores were also statistically comparable across groups (all *p* > 0.05), indicating adequate baseline homogeneity prior to the educational intervention.

**Table 1:** Baseline characteristics and group homogeneity.

Variable	Spanish-audio (*n* = 55)	Quechua-audio (*n* = 58)	Spanish-video (*n* = 60)	Quechua-video (*n* = 54)	Total (*n* = 227)	*p*-value
Sociodemographic characteristics						
Age, median (IQR)	9 (9–11)	10 (8.8–11.0)	10 (9–11)	10 (9–11)	10 (9–11)	0.430
Sex, *n* (%)						0.774
Male	24 (43.6)	30 (51.7)	26 (43.3)	24 (44.4)	104 (45.8)	
Female	31 (56.4)	28 (48.3)	34 (56.7)	30 (55.6)	123 (54.2)	
Maternal education, *n* (%)						0.113
Primary	7 (12.7)	10 (17.2)	18 (30.0)	12 (22.2)	47 (20.7)	
Secondary	44 (80.0)	42 (72.4)	33 (55.0)	33 (61.1)	152 (67.0)	
Higher education	4 (7.3)	6 (10.3)	9 (15.0)	9 (16.7)	28 (12.3)	
Number of siblings, median (IQR)	2 (1–3)	2 (0.0–3.3)	2 (1–3)	2 (0–3)	2 (1–3)	0.860
Baseline outcomes						
Knowledge score, median (IQR)	3 (2–4)	3 (2–4)	3 (3–4)	3 (2–4)	3 (2–4)	0.767
Attitudes score, median (IQR)	21 (19–23)	19 (18–25)	21 (19.3–24.0)	19 (18–24)	20 (18–24)	0.571
Behaviors score, median (IQR)	21 (17–22)	21 (16.0–21.3)	19 (17.3–24.0)	19 (16–22)	20 (17–22)	0.161

IQR, interquartile range. Data are presented as median (IQR) or *n* (%). Group comparisons were performed using the Chi-square test for categorical variables and the Kruskal–Wallis test for continuous variables.

### 3.3 Longitudinal changes in knowledge

Knowledge scores increased significantly over time in all intervention groups (Friedman test, all *p* < 0.001) (Table 2). At baseline, knowledge scores were comparable across groups, with median values ranging from 3 (IQR: 2–4) to 3 (IQR: 3–4), and no significant between-group differences were observed (*p* = 0.767).

**Table 2:** Longitudinal changes in knowledge scores according to intervention group.

Time	Spanish-audio (*n* = 55)	Quechua-audio (*n* = 58)	Spanish-video (*n* = 60)	Quechua-video (*n* = 54)	Between-group *p*-value
Baseline, median (IQR)	3 (2–4)	3 (2–4)	3 (3–4)	3 (2–4)	0.767
Immediate post-test, median (IQR)	4 (3–4)	4 (4–5)	4 (4–5)	4 (4–5)	<0.001
24-h follow-up, median (IQR)	4 (3–5)	4 (3–5)	4 (3–5)	4 (4–5)	0.032
7-day follow-up, median (IQR)	4 (3–5)	5 (4–5)	4 (4–5)	5 (4–5)	0.050
Within-group Friedman test *p*-value	<0.001	<0.001	<0.001	<0.001	–

IQR, interquartile range. Data are presented as median (IQR). Between-group comparisons were performed using the Kruskal–Wallis test, and within-group longitudinal changes were assessed using the Friedman test.

Immediately after the intervention, significant between-group differences emerged (*p* < 0.001). The Quechua-Audio, Spanish-Video, and Quechua-Video groups achieved median scores of 4 (IQR: 4–5), whereas the Spanish-Audio group reached a median score of 4 (IQR: 3–4).

At the 24-hour follow-up, between-group differences remained statistically significant (*p* = 0.032). The Quechua-Video group showed the highest score distribution, with a median score of 4 (IQR: 4–5), while the remaining groups presented median scores of 4 (IQR: 3–5).

At the 7-day follow-up, the overall between-group comparison yielded a *p*-value of 0.050. Descriptively, both Quechua-language groups achieved median knowledge scores of 5 (IQR: 4–5), whereas the Spanish-language groups maintained median scores of 4 (IQR: 3–5 and 4–5, respectively). However, no pairwise between-group differences remained statistically significant after Bonferroni correction.

**Figure 2:**
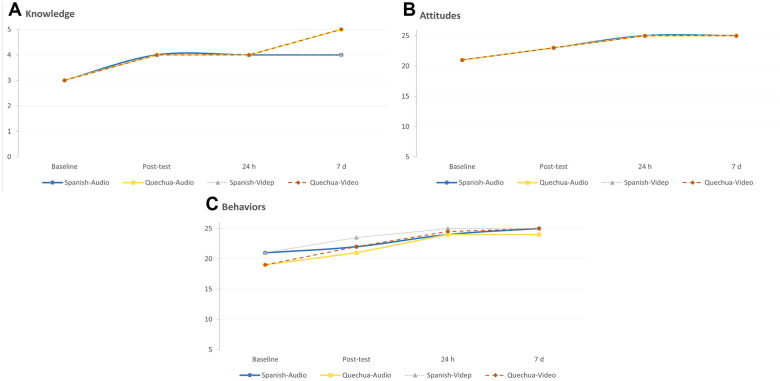
Longitudinal changes in oral health knowledge **(A)**, attitudes **(B)**, and behaviors **(C)** among schoolchildren according to intervention modality and language.

Post hoc analyses confirmed significant improvements from baseline to the immediate post-test, 24-hour follow-up, and 7-day follow-up assessments in all intervention groups (Supplementary Table S1). Figure 2A illustrates that the greatest descriptive separation in median knowledge scores occurred at the 7-day follow-up, with both Quechua-language interventions showing higher median values than the Spanish-language interventions. However, these descriptive differences should be interpreted cautiously because pairwise comparisons were not statistically significant after adjustment for multiple testing.

### 3.4 Longitudinal changes in attitudes

Attitude scores increased significantly over time in all intervention groups (Friedman test, all *p* < 0.001) (Table 3). At baseline, median attitude scores were comparable among groups, with no significant between-group differences observed (*p* = 0.571).

**Table 3:** Longitudinal changes in attitudes.

Time	Spanish-Audio (*n* = 55)	Quechua-Audio (*n* = 58)	Spanish-Video (*n* = 60)	Quechua-Video (*n* = 54)	Between-group *p*-value
Baseline, median (IQR)	21 (19–23)	21 (18–25)	21 (19.3–24.0)	21 (18–24)	0.571
Immediate post-test, median (IQR)	23 (21–25)	23 (22–25)	23 (21–25)	23 (21–25)	0.458
24-h follow-up, median (IQR)	25 (22–25)	25 (21–25)	25 (21–25)	25 (21–25)	0.928
7-day follow-up, median (IQR)	25 (24–25)	25 (22–25)	25 (22.3–25)	25 (22.8–25.0)	0.386
Within-group Friedman test *p*-value	<0.001	<0.001	<0.001	<0.001	–

IQR, interquartile range. Data are presented as median (IQR). Between-group comparisons were performed using the Kruskal–Wallis test, and within-group longitudinal changes were assessed using the Friedman test.

Immediately after the intervention, median attitude scores increased from 21 points at baseline to 23 points across all groups. Further improvements were observed at the 24-hour follow-up, when all groups reached a median score of 25 points. These gains were maintained at the 7-day follow-up, with median scores remaining at 25 points in all intervention groups.

No significant between-group differences were detected at any assessment time point (all *p* > 0.05), indicating that the four educational strategies produced similar improvements in attitudes toward oral health.

Post hoc analyses demonstrated significant improvements from baseline to all subsequent assessments in every intervention group (Supplementary Table S2). Additional gains between post-intervention assessments were observed in some groups, particularly in the Spanish-Audio, Spanish-Video, and Quechua-Video interventions, suggesting a progressive consolidation of positive attitudes over time. Longitudinal trajectories are presented in Figure 2B.

### 3.5 Longitudinal changes in behaviors

Behavior scores improved significantly over time in all intervention groups (Friedman test, all *p* < 0.001) (Table 4). At baseline, median behavior scores ranged from 19 to 21 points, with no significant differences observed among groups (*p* = 0.161).

**Table 4:** Longitudinal changes in behaviors.

Time	Spanish-Audio (*n* = 55)	Quechua-Audio (*n* = 58)	Spanish-Video (*n* = 60)	Quechua-Video (*n* = 54)	Between-group *p*-value
Baseline, median (IQR)	21 (17–22)	19 (16–21.3)	21 (17.3–24)	19 (16–22)	0.161
Immediate post-test, median (IQR)	22 (21–24)	21 (19–25)	23.5 (20–25)	22 (18.8–25)	0.172
24-h follow-up, median (IQR)	24 (21–25)	24 (20.8–25)	25 (21–25)	24.5 (21–25)	0.582
7-day follow-up, median (IQR)	25 (22–25)	24 (21–25)	25 (22–25)	25 (22–25)	0.264
Within-group Friedman test *p*-value	<0.001	<0.001	<0.001	<0.001	–

IQR, interquartile range. Data are presented as median (IQR). Between-group comparisons were performed using the Kruskal–Wallis test, and within-group longitudinal changes were assessed using the Friedman test.

Immediately after the intervention, behavior scores increased across all intervention groups, with median values ranging from 21 to 23.5 points. Additional improvements were observed at the 24-hour follow-up, when median scores reached 24–25 points. These gains were maintained at the 7-day follow-up, with median scores ranging from 24 to 25 points.

No statistically significant between-group differences were identified at any assessment time point (all *p* > 0.05), indicating that the four educational strategies produced comparable improvements in oral health-related behaviors.

Post hoc analyses confirmed significant improvements from baseline to all subsequent assessments in every intervention group (Supplementary Table S3). Additional improvements between post-intervention assessments were observed in the Quechua-Audio and Quechua-Video groups, whereas behavior scores in the Spanish-Audio and Spanish-Video groups tended to stabilize after the initial increase. Longitudinal trajectories are presented in Figure 2C.

### 3.6 Between-group comparisons in knowledge

Pairwise between-group comparisons of knowledge scores are presented in Supplementary Table S4. No significant differences were observed among intervention groups at baseline, confirming the comparability of participants before the educational intervention.

Immediately after the intervention, the Spanish-Audio group showed significantly lower knowledge scores than the Quechua-Audio (U = 938.0, *p* < 0.001, r = 0.374), Spanish-Video (U = 1,098.5, *p* = 0.001, r = 0.302), and Quechua-Video groups (U = 853.0, *p* < 0.001, r = 0.385). These differences corresponded to small-to-moderate effect sizes.

At the 24-hour follow-up, a significant difference remained only between the Spanish-Audio and Quechua-Video groups (U = 1,060.0, *p* = 0.007, r = 0.260), representing a small effect size.

No pairwise differences remained statistically significant after Bonferroni correction at the 7-day follow-up. Likewise, no significant differences were observed between the Quechua-Audio, Spanish-Video, and Quechua-Video groups at any assessment point.

Overall, the findings suggest that the Spanish-Audio intervention showed comparatively lower short-term knowledge gains during the early post-intervention assessments. However, these differences became less evident over time, and no pairwise between-group differences remained statistically significant after adjustment for multiple comparisons at the 7-day follow-up.

### 3.7 Effect sizes of longitudinal changes

The magnitude of longitudinal changes within each intervention group was assessed using Kendall's coefficient of concordance (W) derived from the Friedman test (Supplementary Table S5).

For knowledge outcomes, effect sizes ranged from moderate to large. Large effects were observed in the Quechua-Audio (W = 0.503) and Quechua-Video groups (W = 0.574), whereas moderate effects were found in the Spanish-Audio (W = 0.361) and Spanish-Video groups (W = 0.468).

For attitudes, effect sizes ranged from small to moderate. Moderate effects were identified in the Spanish-Audio (W = 0.383) and Quechua-Video groups (W = 0.349), while small effects were observed in the Quechua-Audio (W = 0.158) and Spanish-Video groups (W = 0.254).

For behaviors, a moderate effect was observed in the Quechua-Video group (W = 0.469), whereas the Spanish-Audio (W = 0.201), Quechua-Audio (W = 0.290), and Spanish-Video groups (W = 0.255) demonstrated small effect sizes.

Overall, the largest longitudinal effects were observed for knowledge outcomes, followed by attitudes and behaviors, indicating that the educational interventions produced their strongest impact on knowledge acquisition and retention.

## 4 Discussion

The present study evaluated the effectiveness of culturally adapted oral health educational interventions delivered through audio and video modalities in Spanish and Quechua among rural schoolchildren from Quechua-speaking communities. All intervention strategies were associated with significant improvements in oral health knowledge, attitudes, and behaviors throughout the study period. However, differences emerged in knowledge acquisition, as participants exposed to the Quechua-Audio, Spanish-Video, and Quechua-Video interventions achieved greater short-term gains than those receiving the Spanish-Audio intervention. In addition, knowledge outcomes showed the largest longitudinal effect sizes, whereas changes in attitudes and behaviors were comparatively smaller. Overall, these results indicate that culturally adapted educational interventions can enhance oral health learning among rural schoolchildren. The comparatively lower performance observed in the Spanish-Audio group also suggests that educational effectiveness may be influenced by the interaction between language adaptation and delivery format, although the independent contribution of each component cannot be determined from the present study design.

The higher knowledge scores observed among children who received the Quechua-language interventions suggest that language may have contributed to facilitating comprehension and assimilation of the educational content, particularly in a population where Quechua remains the primary language of everyday communication. Previous research has shown that educational interventions delivered in participants’ native or culturally familiar languages can enhance understanding, increase engagement, and improve the uptake of health-related information (17, 25). A large scoping review examining Indigenous language vitality and health across Australia, Canada, New Zealand, and the United States found that Indigenous-language use was consistently associated with positive outcomes in health promotion, education, well-being, and community participation, supporting the notion that language functions as an important social determinant of health (17). Similarly, evidence from bilingual education research suggests that instruction delivered in a child's first language may offer cognitive and literacy-related advantages that support knowledge acquisition (26).

During the COVID-19 pandemic in Peru, Indigenous organizations developed radio campaigns in Quechua to communicate public health information to rural communities. These initiatives improved community participation and demonstrated the importance of delivering health messages in the language most familiar to the target population (12). Likewise, previous oral health research has emphasized that linguistic barriers and limited intercultural adaptation contribute to oral health inequalities among Indigenous populations (18). In the present study, both Quechua-language interventions reached the highest median knowledge scores at the 7-day follow-up, and the largest longitudinal effects were observed in the Quechua-Audio and Quechua-Video groups. Nevertheless, pairwise between-group differences at this time point were not statistically significant after adjustment for multiple comparisons. Therefore, these findings should be interpreted as descriptive patterns that warrant confirmation in studies with longer follow-up periods and larger number of clusters.

The educational delivery modality may also have contributed to the differences observed in knowledge acquisition. Although the study was not designed to isolate the independent effects of language and format, participants in the Spanish-Video and Quechua-Video groups consistently achieved knowledge gains comparable to those observed in the Quechua-Audio group and greater than those obtained in the Spanish-Audio intervention immediately after the educational sessions. The better performance observed in the video-based interventions may be related to the simultaneous presentation of visual and verbal information, which can facilitate understanding and retention of educational content among children (19, 26). Previous studies have reported similar findings. In Indonesia, oral health education delivered through animated cartoons increased children's knowledge about toothbrushing practices (27), whereas animated video programs were associated with improvements in oral health behaviors among primary school students (20). Comparable results have been reported in preschool populations, where children exposed to animated video education achieved better oral hygiene outcomes and lower plaque levels than those receiving conventional instruction (28). More recently, educational interventions based on edutainment have shown favorable effects on oral health behaviors and oral hygiene indicators during follow-up periods (29). Although the present study did not directly evaluate the mechanisms responsible for these differences, the findings suggest that incorporating visual resources into educational materials may enhance children's learning experiences and contribute to greater short-term knowledge gains.

The favorable results observed in the Quechua-language and video-based interventions may also be related to the cultural adaptation of the educational materials. Evidence from different settings suggests that educational resources are often better received when they reflect the language and cultural context of the target population. Among Nigerian schoolchildren, a culturally adapted oral health video presented in the Yoruba language produced significant improvements in oral hygiene indicators (30). Likewise, oral health programs developed for Indigenous and socially vulnerable communities have reported positive educational outcomes when local language, cultural traditions, and community values were incorporated into the intervention design (31, 32). These observations are consistent with broader evidence indicating that culturally meaningful educational strategies facilitate participant engagement and strengthen the effectiveness of health promotion initiatives in minority and Indigenous populations (18, 25). Collectively, these findings suggest that educational effectiveness depends not only on the transmission of information but also on the extent to which educational resources resonate with the sociocultural realities of the target population. This may help explain why the interventions incorporating Quechua language and/or visual components achieved the most favorable knowledge outcomes in the present study.

Despite the differences observed in knowledge outcomes, no significant between-group differences were identified for attitudes or behaviors at any assessment point. All four educational strategies produced significant improvements over time, and participants reached similarly favorable scores during follow-up assessments. This finding suggests that, although language and educational modality may influence the acquisition and retention of oral health knowledge, their impact on attitudes and self-reported behaviors may be less pronounced in the short term. Oral health attitudes and behaviors are shaped not only by educational exposure but also by family practices, social norms, environmental conditions, access to resources, and opportunities to apply newly acquired knowledge in everyday life (19, 33). Consequently, behavioral changes often require longer periods of reinforcement and repeated exposure than knowledge acquisition alone (19, 31). This interpretation is consistent with evidence indicating that improvements in oral health literacy do not always translate immediately into measurable behavioral changes, particularly in socially vulnerable populations (33).

The improvements observed in all four intervention groups are consistent with findings reported in previous oral health education studies. For example, a preventive educational strategy implemented among primary schoolchildren in Ciego de Ávila, Cuba, resulted in significant gains in oral health knowledge following the intervention (34). Comparable results have been reported among adolescents participating in structured oral health literacy programs, where improvements were observed in cognitive, communicative, behavioral, and media-related literacy domains (19). Educational initiatives designed for Indigenous populations have also documented increases in oral health awareness, self-efficacy, and preventive practices after community-based activities adapted to local contexts (31). From a programmatic perspective, these findings suggest that relatively brief, culturally adapted educational interventions may achieve meaningful short-term improvements in oral health literacy without requiring complex technological or financial resources, making them particularly suitable for rural and underserved school settings where access to culturally appropriate oral health education is often limited.

Not all outcomes changed to the same extent. In the present study, the largest longitudinal effects were observed in the knowledge domain, particularly in the Quechua-Audio and Quechua-Video groups, whereas attitudes and behaviors showed more modest effect sizes. This pattern was evident even though statistically significant improvements were detected across all three domains. By the 7-day follow-up, knowledge scores had reached their highest levels, while changes in attitudes and behaviors were less pronounced. Similar patterns have been described in previous oral health education studies, where knowledge tends to improve shortly after exposure to educational content, whereas changes in attitudes and everyday practices usually emerge more gradually (19, 29, 31). One possible explanation is that behavioral change depends on factors beyond the educational intervention itself, including family influence, school environments, access to resources, and opportunities to apply preventive recommendations in daily life (19, 33). Research on health literacy and behavior change also suggests that adopting new habits often requires reinforcement over time rather than a single educational experience (29, 33). From this perspective, the stronger effects observed for knowledge may reflect an earlier stage in the learning process, while changes in attitudes and behaviors may become more evident only after longer periods of observation.

Whether these short-term improvements translate into sustained behavioral changes and long-term oral health benefits remains to be determined.

Although the present study was not designed to evaluate clinical oral health outcomes, the magnitude of the observed improvements has practical implications for school-based oral health promotion. The moderate-to-large longitudinal effect sizes observed for knowledge, particularly among children receiving the culturally adapted Quechua-language interventions, suggest that the intervention produced educational improvements that are not only statistically detectable but also potentially meaningful for school-based health promotion.through studies with extended follow-up. Nevertheless, translating these educational gains into measurable improvements in oral health status will probably require ongoing reinforcement through comprehensive school-based oral health promotion strategies.

These findings acquire particular relevance in rural Quechua-speaking communities, where oral health problems remain common despite ongoing school-based oral health promotion efforts. In remote communities of the Cusco region, Bergeron et al. (35) reported very high levels of dental plaque and dental caries among schoolchildren, indicating that existing preventive strategies have not fully addressed the burden of oral disease. Against this background, the results of the present study suggest that culturally adapted educational approaches can improve knowledge, attitudes, and behaviors among children living in rural Indigenous settings. Although the present investigation did not evaluate clinical oral health outcomes, the observed improvements in educational indicators represent an important prerequisite for the adoption of healthier oral hygiene practices and may support future preventive strategies aimed at reducing oral health inequalities in rural Indigenous populations (31, 33).

### 4.1 Strengths and limitations

The present study has several strengths that enhance the relevance of its findings. It addresses a notable gap in the oral health literature by evaluating culturally adapted educational interventions among Quechua-speaking rural schoolchildren, a population that remains underrepresented in oral health research. The inclusion of both linguistic adaptation (Quechua vs. Spanish) and educational delivery modality (audio vs. video) allowed a more comprehensive assessment of factors that may influence learning outcomes in culturally diverse settings. In addition, repeated assessments conducted immediately after the intervention and at follow-up provided valuable information regarding short-term knowledge retention and changes in attitudes and behaviors. Furthermore, the study provides evidence from an underrepresented educational setting in the international oral health literature.

The findings should be interpreted in light of several methodological considerations. The seven-day follow-up period allowed assessment of short-term changes but does not permit conclusions regarding the persistence of the observed effects over longer periods. This aspect is particularly relevant because modifications in attitudes and daily health practices may require sustained reinforcement before becoming established. The behavioral and attitudinal outcomes were measured through self-report, which may not always correspond to actual practices. In addition, the study evaluated educational outcomes without incorporating clinical measures such as dental plaque, gingival status, or dental caries, making it impossible to determine whether the observed improvements translated into measurable oral health benefits. Another factor that warrants consideration is that individual proficiency in Quechua and Spanish was not formally assessed; therefore, differences in language competence could not be examined as a potential influence on educational outcomes. Finally, the study was conducted in a specific rural Andean context, and caution is required when extrapolating the findings to Indigenous populations with different linguistic, cultural, or educational backgrounds.

An additional methodological consideration relates to the partially clustered design. Language allocation was performed at the classroom level, whereas the educational modality was assigned individually within classrooms. Because only four classroom clusters were available, analyses accounting for clustering through multilevel models or generalized estimating equations were not considered statistically reliable. Consequently, although the individual-level analyses were appropriate for the implemented allocation strategy, some underestimation of standard errors associated with the clustered language allocation cannot be completely excluded.

In addition, longitudinal analyses were based on complete-case data because only participants with complete follow-up assessments were included. Although the proportion of missing data was small, excluding participants with incomplete follow-up may have introduced a limited risk of attrition bias.

### 4.2 Implications for oral health promotion and future research

The differences observed between educational modalities indicate that several aspects of intervention design deserve further investigation. In particular, the relative contribution of language, audiovisual presentation, and storytelling elements remains unclear because these components were evaluated simultaneously. Extending follow-up periods and incorporating clinical indicators such as dental plaque, gingival status, or caries experience would help determine whether the improvements observed in knowledge, attitudes, and behaviors are maintained over time and accompanied by measurable oral health benefits. The findings also suggest that oral health promotion activities developed for Quechua-speaking communities may benefit from the use of locally relevant languages and educational materials that reflect the cultural context of the target population. Within rural school settings, these adaptations could facilitate communication and strengthen children's engagement with preventive oral health messages.

## 5 Conclusion

The present findings indicate that culturally and linguistically adapted oral health education can be successfully implemented in Quechua-speaking rural school settings using either audio or video resources. Improvements were observed across knowledge, attitudes, and behaviors, with the greatest gains occurring in oral health knowledge. The Quechua-language interventions consistently demonstrated the most favorable descriptive knowledge outcomes, although all educational approaches were associated with positive changes during follow-up.

These findings support the incorporation of culturally and linguistically appropriate educational strategies into school-based oral health promotion programs serving Indigenous and other culturally diverse communities, where contextually relevant educational approaches may contribute to reducing oral health inequalities.

## Data Availability

The raw data supporting the conclusions of this article will be made available by the authors, without undue reservation.
