# Supplementary material for: Comparative effectiveness of culturally adapted oral health education in rural Quechua-speaking schoolchildren: a cluster quasi-experimental study
Source: Front Oral Health. 2026 Jul 31;7:1903080. doi: 10.3389/froh.2026.1903080 (PMC13472993; doi:10.3389/froh.2026.1903080)
Supplement: Supplementary file 1 [file Table1.docx]

Supplementary Material

**Supplementary Table 1.** Pairwise post hoc comparisons of knowledge scores across assessment time points using the Wilcoxon signed-rank test with Bonferroni correction.

| **Comparison** | **Spanish-Audio p-value** | **Quechua-Audio p-value** | **Spanish-Video p-value** | **Quechua-Video p-value** |
| --- | --- | --- | --- | --- |
| Immediate post-test vs Baseline | <0.001* | <0.001* | <0.001* | <0.001* |
| 24 h vs Baseline | <0.001* | <0.001* | <0.001* | <0.001* |
| 7 days vs Baseline | <0.001* | <0.001* | <0.001* | <0.001* |
| 24 h vs Immediate post-test | 0.080 | 0.442 | 0.685 | 0.861 |
| 7 days vs Immediate post-test | <0.001* | 0.088 | 0.136 | 0.028 |
| 7 days vs 24 h | <0.001* | 0.002* | 0.007* | 0.001* |

*Statistically significant after Bonferroni correction (adjusted significance threshold: p < 0.0083).

**Supplementary Table 2**. Pairwise post hoc comparisons of attitudes scores across assessment time points using the Wilcoxon signed-rank test with Bonferroni correction.

| **Comparison** | **Spanish-Audio p-value** | **Quechua-Audio p-value** | **Spanish-Video p-value** | **Quechua-Video p-value** |
| --- | --- | --- | --- | --- |
| Immediate post-test vs Baseline | <0.001* | <0.001* | <0.001* | <0.001* |
| 24 h vs Baseline | <0.001* | <0.001* | <0.001* | <0.001* |
| 7 days vs Baseline | <0.001* | <0.001* | <0.001* | <0.001* |
| 24 h vs Immediate post-test | 0.003* | 0.288 | 0.131 | 0.087 |
| 7 days vs Immediate post-test | <0.001* | 0.056 | 0.004* | <0.001* |
| 7 days vs 24 h | <0.001* | 0.116 | <0.001* | 0.005* |

*Statistically significant after Bonferroni correction (adjusted significance threshold: p < 0.0083).

**Supplementary Table 3.** Pairwise post hoc comparisons of behaviors scores across assessment time points using the Wilcoxon signed-rank test with Bonferroni correction.

| **Comparison** | **Spanish-Audio p-value** | **Quechua-Audio p-value** | **Spanish-Video p-value** | **Quechua-Video p-value** |
| --- | --- | --- | --- | --- |
| Immediate post-test vs Baseline | <0.001* | <0.001* | <0.001* | <0.001* |
| 24 h vs Baseline | <0.001* | <0.001* | <0.001* | <0.001* |
| 7 days vs Baseline | <0.001* | <0.001* | <0.001* | <0.001* |
| 24 h vs Immediate post-test | 0.021 | 0.006* | 0.151 | <0.001* |
| 7 days vs Immediate post-test | 0.004* | <0.001* | 0.006* | <0.001* |
| 7 days vs 24 h | 0.119 | 0.004* | 0.019 | <0.001* |

*Statistically significant after Bonferroni correction (adjusted significance threshold: p < 0.0083).

**Supplementary Table 4.** Between-group pairwise comparisons of knowledge scores across post-intervention assessment time points.

| Comparison | Time point | U | Z | p-value | r |
| --- | --- | --- | --- | --- | --- |
| Spanish-Audio vs Quechua-Audio | Immediate post-test | 938.0 | -3.974 | <0.001* | 0.374 |
| Spanish-Audio vs Quechua-Audio | 24-h follow-up | 1198.5 | -2.399 | 0.016 | 0.226 |
| Spanish-Audio vs Quechua-Audio | 7-day follow-up | 1280.0 | -2.014 | 0.044 | 0.189 |
| Spanish-Audio vs Spanish-Video | Immediate post-test | 1098.5 | -3.234 | 0.001* | 0.302 |
| Spanish-Audio vs Spanish-Video | 24-h follow-up | 1345.5 | -1.787 | 0.074 | 0.167 |
| Spanish-Audio vs Spanish-Video | 7-day follow-up | 1530.5 | -0.724 | 0.469 | 0.068 |
| Spanish-Audio vs Quechua-Video | Immediate post-test | 853.0 | -4.019 | <0.001* | 0.385 |
| Spanish-Audio vs Quechua-Video | 24-h follow-up | 1060.0 | -2.715 | 0.007* | 0.26 |
| Spanish-Audio vs Quechua-Video | 7-day follow-up | 1151.0 | -2.260 | 0.024 | 0.216 |
| Quechua-Audio vs Spanish-Video | Immediate post-test | 1586.5 | -0.893 | 0.372 | 0.082 |
| Quechua-Audio vs Spanish-Video | 24-h follow-up | 1637.5 | -0.591 | 0.555 | 0.054 |
| Quechua-Audio vs Spanish-Video | 7-day follow-up | 1481.5 | -1.56 | 0.119 | 0.144 |
| Quechua-Audio vs Quechua-Video | Immediate post-test | 1511.0 | -0.35 | 0.727 | 0.033 |
| Quechua-Audio vs Quechua-Video | 24-h follow-up | 1511.5 | -0.342 | 0.732 | 0.032 |
| Quechua-Audio vs Quechua-Video | 7-day follow-up | 1525.5 | -0.277 | 0.782 | 0.026 |
| Spanish-Video vs Quechua-Video | Immediate post-test | 1429.5 | -1.167 | 0.243 | 0.109 |
| Spanish-Video vs Quechua-Video | 24-h follow-up | 1471.5 | -0.903 | 0.367 | 0.085 |
| Spanish-Video vs Quechua-Video | 7-day follow-up | 1327.5 | -1.87 | 0.061 | 0.175 |

U, Mann–Whitney U statistic; Z, standardized test statistic; r, effect size calculated as |Z|/√N. *Statistically significant after Bonferroni correction (adjusted significance threshold: p < 0.0083).

**Supplementary Table 5.** Effect sizes (Kendall's W) for longitudinal changes in knowledge, attitudes, and behaviors according to intervention group.

| **Outcome** | **Group** | **N** | **χ²** | **df** | **p-value** | **Kendall's W** |
| --- | --- | --- | --- | --- | --- | --- |
| Knowledge | Spanish-Audio | 55 | 59.529 | 3 | <0.001 | 0.361 |
|  | Quechua-Audio | 58 | 87.580 | 3 | <0.001 | 0.503 |
|  | Spanish-Video | 60 | 84.180 | 3 | <0.001 | 0.468 |
|  | Quechua-Video | 54 | 92.897 | 3 | <0.001 | 0.574 |
| Attitudes | Spanish-Audio | 55 | 63.275 | 3 | <0.001 | 0.383 |
|  | Quechua-Audio | 58 | 27.422 | 3 | <0.001 | 0.158 |
|  | Spanish-Video | 60 | 45.777 | 3 | <0.001 | 0.254 |
|  | Quechua-Video | 54 | 56.593 | 3 | <0.001 | 0.349 |
| Behaviors | Spanish-Audio | 55 | 33.164 | 3 | <0.001 | 0.201 |
|  | Quechua-Audio | 58 | 50.489 | 3 | <0.001 | 0.290 |
|  | Spanish-Video | 60 | 45.865 | 3 | <0.001 | 0.255 |
|  | Quechua-Video | 54 | 75.955 | 3 | <0.001 | 0.469 |

χ², Friedman chi-square statistic; df, degrees of freedom; W, Kendall's coefficient of concordance. Effect size interpretation: W < 0.10, negligible; 0.10–0.29, small; 0.30–0.49, moderate; ≥0.50, large.
